# Life cycle inventory and assessment data for quantifying the environmental impacts of a wide range of food products belonging to the same food category: A case study of 80 pizzas representatives of the French retail market

**DOI:** 10.1016/j.dib.2022.107950

**Published:** 2022-02-13

**Authors:** Adeline Cortesi, Caroline Pénicaud, Anne Saint-Eve, Louis-Georges Soler, Isabelle Souchon

**Affiliations:** a Université Paris-Saclay, INRAE, AgroParisTech, UMR SayFood, F-78850, Thiverval-Grignon, France; b Université Paris-Saclay, INRAE, UR ALISS, 94205, Ivry-sur-Seine, France; c Université d'Avignon, INRAE, UMR SQPOV, 84911 Avignon Cedex 9, France

**Keywords:** Life cycle assessment (LCA), Food industry, Supply chain, Processed food, Recipe

## Abstract

Most of the time, Life Cycle Assessments (LCA) of food products are performed only on one representative of a food category. This doesn't allow us to understand the possible variations of environmental impacts within a food product category and the responsible factors for these variations. For this reason, LCAs were conducted for 80 different industrial pizzas representative of the French retail market. The LCAs were performed using the “EF 3.0 Method (adapted) V1.00/EF 3.0 normalization and weighting set” on SimaPro software. Most of the data used were taken from the AGRIBALYSE 3.0 and Ecoinvent 3.6 databases. The system perimeter goes from the production of the ingredients to the pizza consumption. The functional unit used was 1 kg of ready-to-eat pizza. Life cycle inventories were made to include the different flows in the LCA (materials, transport, energy, water, waste, etc.). The dataset contains details on products, life-cycle inventories (LCI) and LCIA results. These data can enrich the discussion on the need to study the environmental impacts of different products belonging to the same food category and not only one representative in order to avoid erroneous conclusions.

## Specifications Table

**Table:** 

Subject	Environmental Engineering
Specific subject area	Environmental assessment in the food industry
Type of data	Table
How data were acquired	Inventory data were found in scientific and technical literature completed by experts’ opinions. Life-cycle assessment using SimaPro software and the « EF 3.0 Method (adapted) V1.00 / EF 3.0 normalization and weighting set » was performed. Data from the AGRIBALYSE 3.0 and Ecoinvent 3.6 databases were mainly used.
Data format	Raw Analysed
Parameters for data collection	80 pizzas representative of the pizza retail market in France in terms of recipes, sectors and distributors were selected. Primary data consisted of information written on the packaging of pizzas and weighting of the packaging. Secondary data was collected from scientific literature, machine data sheets or expert estimates.
Description of data collection	The 80 pizzas were selected among the 392 from the French market available in OQALI database. Pizzas recipes were estimated based on the packaging information: the nutritional compositions and the ingredient lists. The recipe of pizza dough was estimated according to an online recipe. Data related to cheeses were estimated according to internal studies. The consumptions needed for the production of the pizzas (energy, water, materials) were estimated using scientific literature, machine data sheets and expert estimates. The weights of the pizza packaging were measured on a balance. Transportation distances were calculated based on assumptions. Storage times and conditions at the wholesaler and at the point of sale were taken from the DEFRA report [1]. The storage times at the consumer's home are based on assumptions and the electricity consumption data related to storage and cooking are taken from data sheets. AGRIBALYSE 3.0 and Ecoinvent 3.6 databases were used for background LCI. Both these databases were directly available in Simapro software. LCIA data and single scores were calculated by LCA using the ``EF 3.0 Method (adapted) V1.00/EF 3.0 normalization and weighting set'' on SimaPro software.
Data source location	Inventory data from France were selected, when available. In case no such data were available, European data were selected and then global data if no European data were available. All the inventory and calculated data used in this study are stored in INRAE research center in Thiverval-Grignon (FR)
Data accessibility	Repository name: Data INRAE Direct URL to data: https://doi.org/10.15454/IH2ERI
Related research article	Cortesi, A., Pénicaud, C., Saint-Eve, A., Soler, L.G., Souchon, I., Does environmental impact vary widely within the same food category? A case study on industrial pizzas from the French retail market. Journal of Cleaner Production 336 (2022), 130128. https://doi.org/10.1016/j.jclepro.2021.130128

## Value of the Data

• The article presents a unique set of LCI and LCIA data from a set of 80 products from the same food category.
• These data can enrich the discussion on the need to study the environmental impacts of different products belonging to the same food category and not only one representative in order to avoid erroneous conclusions.
• These data can be used as relevant information for assessment of food sector sustainability.
• These data can also be used to make recommendations for more environmentally friendly pizza choices or for pizza reformulation.

## 1 Data Description

The dataset associated with this article contains files containing information, inventory data required for the LCAs and the LCIA results obtained for the 80 pizzas presented in the associated article [2]:

1. dataset_pizzas_nutritional data: nutritional characteristics and composition information on the 80 pizzas: energy, fats, saturated fatty acids, carbohydrates, sugars, fibers, proteins, sodium, dough/topping, mass animal products/mass vegetal products, % cheese, % tomato sauce and vegetables, % dough, % meat and fish.
2. dataset_pizzas_LCI recipes: recipes of the 80 studied pizzas. The recipes are presented for 1 kg of pizza with and without ingredient losses. The percentage of losses for each ingredient is also mentioned in this file.
3. dataset_pizzas_LCI ingredients: name of all the data used for the inventory of the ingredients directly taken from a database and the associated database.
4. dataset_pizzas_LCI other ingredients: inventories of the ingredients not taken directly from a database: dough (made from oil, salt, water, wheat flour and yeast) and cheeses (Asiago, Cheddar, Goat cheese, Comté, Edam, Emmental, Sheep cheese, Melted cheese, Gorgonzola, Gouda, Grana Padano, Maasdam, Mozzarella, Raclette, Roquefort). The names of all the data used for the inventories and the associated database are also given.
5. dataset_pizzas_LCI packaging: weight of the packaging of each of the 80 pizzas studied. The inventory as well as the name and the database of the data used to modelize the packaging is also available.
6. dataset_pizzas_LCI manufacturing: inventory of the flows taken into account for the manufacturing step. The flows included are energy (kWh and MJ), water, wastewater and food waste. The name and the database of the data used to modelize these flows are also mentioned.
7. dataset_pizzas_LCI transports: inventory of the transport from pizza factory to supplier and from supplier to retailer. Assumptions about the distances are provided (km), the total mass of each transported pizza (including packaging, kg) and the Ecoinvent processes used, as well as the type of transport (fresh/frozen) are given.
8. dataset_pizzas_LCI distribution: inventory data related to the storage of the pizzas at the wholesales and at the supermarket for both fresh and frozen pizzas. The flows included are electricity, heat, cooling liquid leak and waste. The Ecoinvent processes used are given.
9. dataset_pizzas_LCI use: electrical consumption of fridge storage and oven cooking for fresh and frozen pizzas for 2 scenarios of storage time. The weights of the packaging wastes taken into account in this step are also available in this file, as well as the names and the database of the data used to modelize the flows.
10. dataset_pizzas_LCIA: LCIA results obtained for the 80 pizzas calculated by the “EF 3.0 Method (adapted) V1.00 / EF 3.0 normalization and weighting set” for the midpoint indicators: Climate change, Ozone depletion, Ionising radiation, Photochemical ozone formation, Particulate matter, Human toxicity, non-cancer, Human toxicity, cancer, Acidification, Eutrophication, freshwater, Eutrophication, marine, Eutrophication, terrestrial, Ecotoxicity, freshwater, Land use, Water use, Resource use, fossils, Resource use, mineral and metals. Results are presented by steps (ingredients, manufacturing, packaging, transport, distribution, use) and in total, for two scenarios: short home storage and long home storage.
11. dataset_pizzas_single score: single scores calculated for the 80 pizzas by the “EF 3.0 Method (adapted) V1.00 / EF 3.0 normalization and weighting set”.

## 2 Experimental Design, Materials and Methods

### 2.1 LCA methodology

Our study followed ISO 14040 [3].

#### 2.1.1 Goal and scope

The goal of the study was to characterize the environmental impact of 80 industrial pizzas from the French retail market (hypermarkets and supermarkets). These pizzas have been chosen to represent the entire French retail market.

#### 2.1.2 System definition

**Fig. 1:**
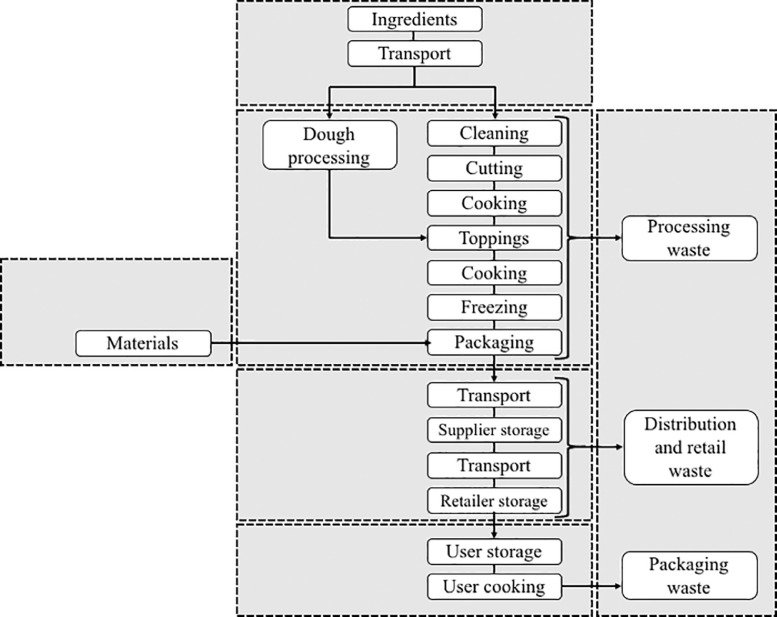
Steps in pizza production considered in the LCA.

Several steps in the life cycle of pizzas were considered (Fig. 1): ingredient production, ingredient transportation, manufacturing of the pizza in the factory, transportation of the pizza, distribution, sale, and domestic use. All the steps mentioned in Fig. 1 are taken into account in the inventory. The waste board and plastic packaging are therefore taken into account.

The flows that were considered were raw materials, water, and energy consumption. Emissions generated by agricultural production of the raw ingredients, packaging materials, electricity, heat and water production, transports as well as emissions due to refrigerant leaks, are already included in the data used (selected in databases). The LCI of the production of the equipment were not taken into account due to their long lifetimes which lead to low environmental impacts when we allocate equipment LCI to its use duration.

Losses, estimated from the ADEME report [4], were considered for pizzas at the retail step. Losses associated with the removal of inedible parts of fresh vegetables used at the manufacturing step were also considered. These various losses are presented in Table 1.

**Table 1:** Percentage of loss for various food products at various steps (from [4]) («NA» means not applicable).

	Manufacturing (%)	Retail (%)
Artichoke	80	NA
Basil	20	NA
Carrot	20	NA
Eggplant	20	NA
Garlic	20	NA
Mushroom	20	NA
Olive	50	NA
Onion	20	NA
Pepper	3	NA
Spinach	3	NA
Zucchini	3	NA
Fresh pizza	NA	5
Frozen pizza	NA	0.6

Losses were summed at each step to calculate the total loss. Thus, the mass of an ingredient considered in the agricultural phase corresponds to the sum of the mass of this ingredient in the final pizza and the losses related to the manufacturing and retail steps.

Losses at the consumer's home were not considered. Similarly, the cleaning of the plant and the transportation of the pizza from the point of sale to the consumer's home were also not considered.

#### 2.1.3 Functional Unit

We chose to use a mass functional unit, which is the functional unit commonly used for the study of the environmental impact of food products. The functional unit used was therefore 1 kg of ready-to-eat pizza (after removing the packaging and cooking) at the consumer's home. We decided to choose a functional unit of 1 kg of pizza ready-to-eat and not at the supermarket because we wanted to evaluate the relative weight of the environmental impact of the use step, including fridge storage and oven cooking. Furthermore, this FU allowed us to compare two scenarios of pizza home storage (short and long durations).

#### 2.1.4 Inventory & data collection

##### 2.1.4.1 Ingredients

###### 2.1.4.1.1 Estimating the proportion of each ingredient in the pizza

The ingredients of each pizza are listed on their packaging in descending order of weight. However, the exact content of each ingredient within a pizza is not mentioned. The nutritional labelling of the pizzas was, thus, used to estimate the mass of each ingredient in the final pizza.

###### 2.1.4.1.2 Databases

The database that was mostly used was AGRIBALYSE 3.0 for agricultural and food products, completed with data from Ecoinvent 3.6 for other data. We used data from the literature for mushrooms [5].

###### 2.1.4.1.3 Cheese

Cheese is an ingredient present in significant quantities in all 80 pizzas studied. Nevertheless, it comes in different forms and 15 different cheeses were identified for all 80 pizzas studied. However, the AGRIBALYSE 3.0 database only distinguishes a few major categories of cheese. Importantly, the environmental impact of a variety of cheeses is highly dependent on the amount of milk used [6], [7], [8]. Ripening time also plays an important role in the overall environmental impact of cheese because of the electricity consumption involved [9]. These two steps can vary greatly from one cheese to another. Thus, the environmental impact of the 15 cheeses is likely to vary. We therefore estimated the inventory data for each cheese by considering that the part related to the process was equivalent for all cheeses and by varying only the quantity of milk required to make the cheese and the ripening period. As part of the milk used to make the cheese is also used to make cream (during skimming (optional step)) and whey during draining, economic allocations were used to determine the portion of environmental impact attributable to the cheese. The masses of cream and whey produced during the manufacture of each cheese were then estimated by attributing the loss of fats to the production of cream and the loss of post-skimming water to the production of whey.

#### 2.1.4.2 Pizza manufacturing

The hypothesis of a relatively automated factory producing an average of 10,000 pizzas of 450 g each day during its 10 hours of daily operation was used. The consumption of the various machines considered for the different steps of pizza production (Table 2) were estimated based on the scientific literature, technical data sheets from the manufacturers of equipment used for the food industry, and expert estimates. The consumption of the machines running continuously was estimated for 10 hours of operation and then reduced to the mass of pizza produced in 10 hours. The consumption of machines considered to not run continuously was estimated for one cycle and then divided by the number of kilograms of pizza processed in one cycle.

**Table 2:** Steps of pizza production considered and associated machines. The machines mentioned may or may not be considered, depending on the pizza.

Step	Machine(s)
Dough preparation	Mixer, elevator/unloader, dough-dividing dough rounder, treadmill
Vegetable preparation	Vegetable washer, cutting machine, cooker
Meat preparation	Cutting machine (cubes or slices), cooker
Cheese preparation	Cutting machine (cubes or slices)
Topping	Filling machines
Cooking	Electric, wood-fired oven
Freezing	Freezing machine
Packaging	Packaging machine, treadmill

#### 2.1.4.3 Packaging

The weights of the plastic and cardboard packaging were measured by weighing them for each of the 80 pizzas using a PRECISA balance (XT 6200C), accuracy = 0.01 g.

#### 2.1.4.4 Transport

Two steps of transport were considered: the transport of the pizza from the factory to the supplier and from the supplier to the retailer. The transport of the pizza from the retailer to the consumers’ homes was not considered. We assumed that the supplier was 1,000 km from the pizza factory and the retailer 200 km from the supplier. Reefer cooling/freezing truck from Ecoinvent 3.6 database was used.

#### 2.1.4.5 Distribution

The DEFRA Report [1] was used to estimate the duration of storage of fresh and frozen pizza at the supplier and the retailer. We considered that fresh pizzas were stored for 12 hours in a refrigerated warehouse at the supplier and 48 hours at the retailer, including 10 hours in a walk-in cooler and 38 hours in a display cabinet. For frozen pizzas, we considered that they were stored for 158 hours in a refrigerated warehouse at the supplier and 120 hours at the retailer, including 24 hours in a walk-in freezer and 96 hours in a display cabinet. The data contained in this report was then used to estimate the electrical consumption and loss of refrigerating liquids associated with these various storage conditions. Electrical consumption related to the lighting of the retailer was also considered. According to Tsarouhas et al. [10], a hypermarket consumes an average of 777kWh of electricity per m^2^ of sales area per year, of which 15 to 25% is directly related to lighting. Volume allocations were made to estimate the consumption linked solely to the storage of 1 kg of pizza for one hour. The values thus calculated were then weighted by the previously estimated duration of storage at the retailer for the two types of pizza.

#### 2.1.4.6 Use

The steps we considered at the users’ homes were storage and baking of the pizza in an electric oven. We hypothesized that the storage was two days for fresh and frozen pizzas. A complementary scenario was also tested to study the influence of the duration of domestic storage on the environmental impact of the pizzas, consisting of a “long” storage scenario, i.e., seven days for fresh pizzas and one year for frozen pizzas. A domestic freezer and refrigerator of equivalent dimensions (200 L and 201 L, respectively) were considered, and their respective electrical consumption was recorded from their data sheets. A volume allocation in these two appliances was made to estimate the electrical consumption solely related to the storage of one kilogram of pizza. The baking times for fresh and frozen pizzas were estimated based on the preparation tips on the pizza packaging. The electrical consumption of a conventional electric oven was defined using a technical data sheet of an appliance and then calculated for the cooking time required to cook 1 kg of pizza.

## Ethics Statement

This work did not involve human subjects or laboratory animal, therefore did not meet any ethical issues.

## Declaration of Competing Interest

The authors declare that they have no known competing financial interests or personal relationships which have, or could be perceived to have, influenced the work reported in this article.

## Data Availability

Dataset on the Life Cycle Assessment of 80 pizzas representatives of the French retail market (Original data) (Dataverse).

## Appendix Supplementary materials

Image, application 1
